# *Lactobacillus sakei*: A Starter for Sausage Fermentation, a Protective Culture for Meat Products

**DOI:** 10.3390/microorganisms5030056

**Published:** 2017-09-06

**Authors:** Monique Zagorec, Marie-Christine Champomier-Vergès

**Affiliations:** 1 Secalim, INRA, LUNAM Université, 44307 Nantes, France; 2 Micalis Institute, INRA, AgroParisTech, Université Paris-Saclay, 78350 Jouy-en-Josas, France; marie-christine.champomier-verges@inra.fr

**Keywords:** meat products, fermentation, biopreservation, spoilage, genomics, diversity, metabolism

## Abstract

Among lactic acid bacteria of meat products, *Lactobacillus sakei* is certainly the most studied species due to its role in the fermentation of sausage and its prevalence during cold storage of raw meat products. Consequently, the physiology of this bacterium regarding functions involved in growth, survival, and metabolism during meat storage and processing are well known. This species exhibits a wide genomic diversity that can be observed when studying different strains and on which probably rely its multiple facets in meat products: starter, spoiler, or protective culture. The emerging exploration of the microbial ecology of meat products also revealed the multiplicity of bacterial interactions *L. sakei* has to face and their various consequences on microbial quality and safety at the end of storage.

## 1. Introduction

First described almost a century ago as a contaminant of the rice wine “saké” [1], *Lactobacillus sakei* is able to colonize many different habitats. It has often been isolated from food of vegetable sources like various flours, sourdoughs, or fermented cabbage [2,3,4,5], but is systematically associated with meat products and often with seafood [6,7,8]. Because of its presence in many foodstuffs, we can therefore consider this lactic acid bacterium (LAB) as belonging to the human diet. Consequently, it has also been isolated from human feces [9,10]. Nevertheless, the gastrointestinal tract does not appear as its preferred environment [10,11]. Indeed, *L. sakei* is the emblematic LAB of meat products—in particular of raw meat products stored at low temperature and under vacuum packaging and of fermented sausages. This results from its metabolic activities and phenotypic traits that are particularly well adapted to its growth and survival under the conditions encountered during meat storage and processing [12]. Since its discovery, many studies have aimed at understanding the physiology of this LAB, focusing on its remarkable adaptation to the meat environment and to optimize its use for biopreservation or as starter for the fermentation of sausages. Several metabolic traits have been described in detail, including its ability to use various nutrients present in meat that confer it a selective advantage. The genomes of several strains have been sequenced [12,13,14,15], and an important intra-species diversity has been revealed [16,17,18,19].

## 2. Adaptation of *L. sakei* to Its Various Habitats

### 2.1. Ability to Use Nutrients Encountered in Meat to Produce Metabolites

#### 2.1.1. Use of Amino-Acids

Meat is a rich substrate providing amino acids and peptides issued from proteolysis of myofibrillar and sarcoplasmic proteins. In *L. sakei*, amino acid prototrophy is strain-dependent [20]. A transcriptomic study showed that in the presence of meat proteins *L. sakei* up-regulated genes encoding oligopeptide transporters and intracellular peptidases [21] indicating the ability of this species to take advantage of meat nutrients. Indeed, the up-regulation of many genes involved in protein translation during growth in the presence of myofiblillar or sarcoplasmic protein extracts, indicated that intracellular amino acids resulting from oligopeptide transport and peptidase activity could be used for *de novo* protein synthesis [21]. These results are in accordance with a proteomic analysis indicating up-regulation of peptidases and proteins involved in translation during growth with meat proteins [22].

The use of arginine, an abundant amino acid present in meat [23], is well documented in *L. sakei*. It involves the arginine deiminase pathway (ADI) that produces ornithine, ammonia, and carbon dioxide and concomitantly generates ATP. In *L. sakei*, the ADI pathway is encoded by the *arcABCTDR*, *PTP* gene cluster [24,25,26]. The transcriptional regulator encoded by *arcR* acts as an activator [25]. Arginine deiminase, ornithine carbamoyl transferase, and carbamate kinase, the three enzymes involved in the ADI pathway, are encoded by *arcA*, *arcB*, and *arcC*, respectively [24]. *arcT* encodes a putative transaminase, and *arcD* the arginine/ornithine antiporter [24]. In addition, the *PTP* gene, located downstream from the *arc* operon, has been shown to be involved in citruline excretion and re-uptake [26]. Arginine utilization by *L. sakei* is submitted to catabolite repression [24], is sensitive to pH [27,28], and is induced by the presence of arginine and by anaerobiosis [29], correlating to conditions encountered during meat storage. Although the use of arginine does not confer an advantage for growth [25], it enhances the survival of *L. sakei* because of the ATP it generates and not as a consequence of ammonia production [29]. Thus, this function participates to *L. sakei* adaptation to meat.

#### 2.1.2. Use of Carbon Sources

Conversely to its richness in amino acids and peptides, meat is a relatively poor substrate for sugars. In laboratory conditions, *L. sakei* strains preferably ferment glucose, *N*-acetyl-d-glucosamine, sucrose, fructose, and mannose, which are transported by the PEP dependent phosphotransferase system (PTS) [12,20]. However, these carbon sources are not very abundant in meat. Ribose is one of the carbohydrates present in raw meat, and *L. sakei* has been shown to use it through an ATP-dependent system [20]. The genes responsible for ribose utilization in *L. sakei* were characterized in [30]. Several uncommon aspects of ribose utilization by *L. sakei* were reported. First, while ribose is transported by ABC transporters in most species, no such ribose transporter was found in *L. sakei*. Ribose uptake was ensured by a ribose transporter suspected to function as a facilitator encoded by the *rbsU* gene, located in the ribose operon [30]. Second, an unusual regulation of ribose utilization was suspected. Whereas utilization of sugars is submitted to catabolite repression by glucose in most species, a fine tuning of the expression of genes involved in carbon catabolism has been suggested in *L. sakei* [31]. In addition, uncommon involvement of the PTS is suspected, although the mechanism has not been elucidated [32]. This is based on the observation that a mutant in *ptsI* (encoding the enzyme I of the PTS) grew faster on ribose than the wild type parent. In this mutant, both ribose uptake and phosphorylation were enhanced, although the transcription of the rbsU and rbsK genes were not overexpressed, suggesting a regulation by the PTS at a posttranscriptional level [30,32].

The gene repertoire of *L. sakei* also suggested the ability of this species to use alternative carbon sources such as nucleosides or *N*-acetyl-neuraminic acid that are present in meat [12]. The utilization of the pentose moiety of adenosine and inosine as a carbon source was confirmed experimentally, proving that *L. sakei* can indeed take advantage of alternative molecules for its carbon catabolism in meat [33]. Additionally, utilization of *N*-acetyl-neuraminic acid through a gene cluster encompassing the *nanTEAR-nanK-lsa1644-lsa1645* genes has been demonstrated [34], and the activity of the *N*-acetylneuraminate lyase encoded by *nanA* has been characterized [35].

A metabolomic study performed in laboratory medium showed that nutrient utilization varies depending on the growth phase: first, *L. sakei* consumed glucose, fructose, carnitine, tryptophan, and malic acid from the medium, and then it used trehalose, citric acid, and lysine that had accumulated into the cells [36]

#### 2.1.3. Other Nutrients

Meat, particularly red meat, is known to be a source of iron, being part of the heme prosthetic group of hemoglobin and myoglobin. The analysis of the genome sequence of *L. sakei* suggested the presence of several transporters putatively involved in iron or heme uptake [12]. As well, *L. sakei* is one of the rare LAB that possess a heme-dependent catalase, although it is unable to synthesize heme [37,38]. Therefore, the ability to transport iron/heme and iron/heme-containing molecules was investigated. The catalase was active when L. sakei cells were cultivated in a medium supplemented with hematin, myoglobin, or hemoglobin, showing that this species could indeed transport heme or heme-carrier molecules [39]. In addition, heminic compounds supplementation of the growth medium enhanced the survival of *L. sakei* during stationary phase. A microscopy approach, based on electron energy loss spectroscopy analysis and nano-scale secondary-ion mass spectrometry, clearly showed that heminic compounds were internalized by *L. sakei*. However, iron (FeCl_3_) did not enter the cells [39].

### 2.2. Ability to Resist to Various Stresses

The analysis of *L. sakei* 23K whole genome sequence revealed the large genetic equipment for resisting harsh conditions encountered during meat storage and meat processing [12]. One of the stresses occurring during meat processing is the oxidative stress that can occur during meat mixing or during storage under oxygen enriched modified atmospheres. The redundancy of genes potentially involved in oxidative stress response (thioredoxins and thioredoxin reductases) and the presence of a putative glutathione/glutaredoxine/glutathione reductase system may contribute to *L. sakei* resistance to oxidative stress. In addition, as mentioned above, *L. sakei* strains possess a heme-dependent catalase, encoded by *katA*, that can be active when heme is present in the medium and transported into the cell. A mangenese/iron superoxide dismutase gene *sodA* is also present in the genome. SodA, in association with the catalase, enables the detoxification against superoxide radicals. Although all *L. sakei* strains harbor a *katA* gene [7,40] the level of resistance to various oxidative stresses is strain-dependent [41,42]. Among lactobacilli, *L. sakei* is one of the best equipped to cope with growth under aerobiosis, a condition that does not lead to improved growth yield, but procures a better tolerance to oxidative stress [43]. In addition, in *L. sakei* mixed cultures, the ability to survive various oxidative stresses also depends on the composition of bacterial population. Indeed, in co-cultures with three different strains, it was shown that some strains may have different behavior depending on the other strains present in the mixtures. As well, some strains may behave as helper or burden to their companions in mixed cultures [42]. Resistance to high salt concentrations that can occur in fermented meats and to the cold temperatures that are of current use for the storage of raw meat products has also been documented. The optimal *L. sakei* growth temperature is 30 °C. Both growth rate and final cell density reached after exponential growth decrease when incubation temperature is lowered. A slow growth is still observed at 4 °C [44]. The combination of low temperature and high NaCl concentration drastically impairs *L. sakei* growth, but acts synergistically and prolongs survival of the cells during stationary phase [44].

The ability to cope with high NaCl concentration, acidic pHs, or high temperatures has been shown to vary between the strains and to involve the expression of regulators from the two-component system family [45].

## 3. Intra-species Diversity of *L. sakei*

### 3.1. The L. sakei Clade

**Figure 1:**
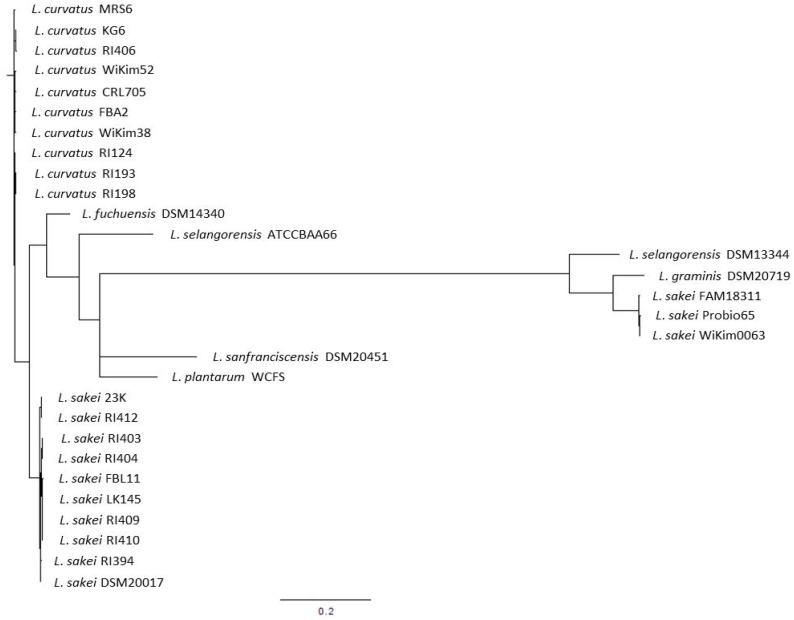
Phylogenetic tree drawn from the *rpoB* gene sequences of the main species belonging to the *L. sakei* clade.

*L. sakei* has been described as closely related to *Lactobacillus curvatus*, *Lactobacillus fuchuensis*, and *Lactobacillus graminis* [46]. These psychrotrophic species are known to be adapted to the meat environment, i.e., to the low temperature of storage used for meat products. The existence of this clade was confirmed through the comparison of the genome sequences available for several strains belonging to these species [47,48]. These comparative analyses also revealed that the closest species to this group was *Lactobacillus selangorensis* initially isolated from Malaysian food [49]. To illustrate this, we have drawn a phylogenetic tree (Figure 1) made from the *rpoB* gene sequence alignment, a method that has been reported as accurate for closely related species and sub-species [50].

We selected *rpoB* genes from 13 *L. sakei*, 10 *L. curvatus*, 1 *L. graminis*, 1 *L. fuchuensis*, and 2 *L. selangorensis* genomes. We used the *rpoB* genes from *Lactobacillus plantarum* and *Lactobacillus sanfranciscensis* as they appeared as the closest out groups. This figure shows clearly the close relatedness of *L. sakei*, *L. curvatus*, and *L. fuchuensis*. However, the two *L. selangorensis rpoB* sequences available supposed to be issued from the same strain (DSM 14340 and ATCC BAA 66) did not cluster together. This might result from sequencing errors as those sequences were obtained from draft genome sequences, as is the case for *L. graminis* DSM 20719 and *L. sakei* Probio65. Surprisingly, an out group was observed that encompassed one *L. selangorensis*, the *L. graminis*, and three *L. sakei* strains. This shows the relatedness of these species and the existence of this clade. However, further analysis would be required to ensure the true clustering of these strains.

Although the *L. sakei* species has been divided into two subspecies (*L. sakei* subsp. *carnosus* and *L. sakei* subsp. *sakei*) based on Random Amplified Polymorphic DNA (RAPD) and phenotypic characterization [51], more recent studies using the detection of strain specific genomic markers or MultiLocus Sequence Typing (MLST) revealed rather several clusters within the species [17,19]. Such heterogeneity of the results obtained by authors aiming at clustering strains with different methods suggested a wide genomic diversity within the species.

### 3.2. The Genomic Diversity among L. sakei Strains

To date, 16 *L. sakei* genome sequences are publicly available [52], with size ranging from 1.88466 to 2.07552 Mb for the complete genomes. A strain-dependent chromosome size variation from ~1.8 to ~2.3 Mb had indeed been reported [16,17,18], suggesting a large variety of strain-specific gene repertoires and therefore a wide phenotypic diversity potential of the species. The first genome sequence of a *L. sakei* strain revealed the presence of genomic islands indicating putative hotspots of gene divergence [12]. This was confirmed in a comparative genomic analysis based on microarrays, which showed that the divergent regions may result from horizontal gene transfer [18]. Consequently, the analysis of the diversity among a large strain collection revealed that a subset of 60 marker genes could be used as a simple PCR tool for classification of *L. sakei* strains into 10 clusters based on the presence or absence of these markers [17]. Among those genes belonging to the flexible gene pool, some may be involved in the fitness of the strains, as various transporter or cell surface component encoding genes [17,18]. Nevertheless, no link could be clearly established between the genome content of the strains and their ecological origin, whatever the method used [16,17,18]. Finally an MLST analysis performed on a large collection of 232 strains showed that the *L. sakei* species was composed of three subpopulations [19].

## 4. *L. sakei:* A Starter for Fermented Sausages

As mentioned above, the species *L. sakei* exhibits properties that ensure it a high fitness for meat environments, especially for fermented ones. Belonging to the indigenous microbiota of meat, it is spontaneously active for the fermentation of artisan sausage. In association with coagulase negative staphylococci, it has been used as a commercial starter for many years for sausage making in different countries worldwide and is especially important in Western Europe [53,54]. A large biodiversity of strains has been reported (see above), and numerous studies have described strain isolation from fermented meat products. The aptitude of starter strains to fast acidification is an important characteristic as it has an impact on taste and safety, aroma, and bacteriostatic and bactericidal effects [53]. Depending on raw materials and indigenous microbial communities, a wide variety of products exist. Besides this main importance in Western Europe, it is noteworthy that *L. sakei* is also involved in fermented meat products in Asia [55], as nem chua in Vietnam [56], or mum, a traditional meat-fermented sausage, in Thailand [57]. *L. sakei* has been also identified in adventitious llama meat fermentation made in the Andean region [58].

Tolerance to high salt concentration and fast acidification may constitute key functions for a significant role of the species in meat fermentation. In order to identify genes involved in meat fermentation, the in vitro expression technology (IVET) was used for exploring the response of a starter strain, *L. sakei* 23K, in a raw sausage model [59]. Fifteen genes were identified whose promoters were specifically induced during meat fermentation, several being associated to stress response. Construction of mutants in some of these genes and assessment of their performance in the meat fermentation model were examined to decipher about their involvement in meat fermentation. Four mutants were altered in their growth, three having a reduced growth (impaired in asparaginase encoding gene, and two unknown function encoding genes) and one (impaired in an heat shock regulator coding gene, *ctsR*) exhibiting enhanced growth. Following this finding of the involvement of *ctsR* gene, it was shown that performance of *L. sakei* in sausage fermentation could be improved by applying stress treatments prior inoculation [60]. Different pre-inoculation treatments (4 °C, 42 °C, or 6% sodium chloride) were applied both on wild type strain and on a *ctsR* mutant. It was in particular noticed an increased growth and acid production for cold stressed cells.

Recently, the effects of ultrasound treatment, a technology being developed in various food engineering processes, have been evaluated on the fermentation capacities of *L. sakei* in a meat model [61]. This revealed that growth stimulation or growth retardation can be obtained by ultrasound treatment depending on the power used. Lactic acid production was not affected by ultrasound treatment. At the opposite antimicrobial activity against *Staphylococcus aureus*, *Listeria monocytogenes*, and *Salmonella* Typhimurium was enhanced in cell-free extracts from treated samples by comparison to untreated control.

Along with their use as starters in association with *Staphylococci* for sausage making, the interactions between these two species have been considered. It has been shown that some of these coagulase negative *Staphylococci* could affect both growth and proteolytic activities of *L. sakei* strains. Such interactions are therefore of interest and should be considered for selection of starters in order to improve the organoleptic properties of fermented sausages [62].

Besides the role of the species in fermentation, *L. sakei* strains isolated from fermented products have also been tested for their health benefits or for their potential use as probiotics to be added in food (See [63,64,65] as recent examples). Resistance to conditions encountered in the gastrointestinal tract (bile salt, pepsin, and acidic pH) was observed as well as the ability to adhere to intestinal cells [64]. Putative health benefits of *L. sakei* as immunomodulatory activity has been reported [65]. A positive effect of extracts of brown rice fermented with *L. sakei* on bone formation has also been reported [63]. However, most of the studies dealing with probiotic or health effect were performed in vitro or using cell cultures or mice models.

## 5. *L. sakei* and Its Use as Protective Culture

Besides its utilization as a starter, mainly based on acidification capacities of the species, the evidenced antibacterial, bacteriostatic, and bactericidal properties of *L. sakei* have prompted researchers and industrials to explore and develop such biopreservation properties in fermented meat and also in fresh meat products. Two main aspects have been considered and studied: the bacteriocinogenic activities that can be effective in fermented or fresh meats and the biopreservation action relying on this property and also on competitiveness allowing biopreservation of fresh meat. Both pathogenic and spoilage bacterial species have been considered as targets for biopreservation in order to increase safety and shelf life of the products.

The most largely explored area has first been the bacteriocin production abilities and their activities against pathogens. A large number of studies have reported bacteriocin production by different *L. sakei* strains and in some cases the molecular mechanisms have been elucidated [66,67]. The ability of protective cultures of interest to develop in fresh meat during their conditioning at low temperature has also been explored through their protein hydrolysis and amino acid production capacities [68]. However, although these reports have deeply characterized the mechanisms of bacteriocin production even at genetic level, the effectiveness of bacteriocin-producing strains and *L. sakei* in food products and particularly in meat appeared to be limited.

Biopreservation trials in fresh meat products have been directed either against pathogens, the main emblematic ones in meat being *L. monocytogenes* and *Escherichia coli*, or against spoilers aiming at extending shelf life. The strategy generally relied on the selection of candidate strains from the targeted products. From sliced cooked ham artificially contaminated with *L. monocytogenes*, indigenous *L. sakei* strains were isolated for their ability to limit the development of this pathogen. These *L. sakei* strains revealed to be able to limit the development of *L. monocytogenes* and *E. coli* O157:H7 [69]. These selected strains have been used successfully as protective cultures with a good consumer acceptance of the so protected hams [70]. Similarly, Vermeiren and coworkers developed strategies in order to set up protective cultures for enhancing cooked ham shelf life and hygienic quality in the 2000s [71,72,73,74]. They selected potential candidates issued from a large screening of meat products and performed co-cultures with such a selected *L. sakei* strain and a bacteriocin-producing strain in a model cooked ham and showed that the bacteriocin producer was inefficient against the spoilage organisms tested (*Leuconostoc mesenteroides* and *Brochothrix thermosphacta*), whereas the selected strain was able to lower their growth.

Fresh-chilled lamb and vacuum-packed beef have also been screened in order to select putative protective cultures [75,76,77]. First, LAB isolated from these products were tested in in vitro models for their ability to limit the growth of selected pathogenic or spoilage bacteria. Six isolates (five *L. sakei* and one *Lactococcus lactis*) appeared to exhibit inhibitory properties either against *B. thermosphacta*, *L. monocytogenes*, or *Clostridium estherteticum* [75]. These selected strains were then successfully tested after inoculation in lamb or beef during prolonged storage at low temperature under vacuum [76]. The best preservation was achieved by using a cocktail of three of these *L. sakei* strains [77]. Such a strategy, relying on strain cocktail development, has later revealed to be promising with *L. sakei* as a protective species. Indeed, in 2014, Chaillou et al. also tested three different *L. sakei* strain cocktails in order to evaluate their ability to inhibit the development of the pathogens *Salmonella* Typhimurium and *E. coli* O 157:H7 and the spoiling species *B. thermosphacta* in ground beef [78]. One of these cocktails was revealed to be efficient by limiting the development of both *S.* Typhimurium and *E. coli*. In cured products such as bacon, *L. sakei* has been reported to be an efficient protective culture, able to limit the spoilage due to the development of *L. mesenteroides* [79,80].

These approaches appear promising for future development of biopreservation for enhanced shelf life and safety of meat products, once the global ecosystems of such products and the complex equilibrium driving their evolution during storage at low temperature are assessed.

Yeasts and molds are not among the most important microorganisms responsible for spoilage of meat products, but they may be of concern in other food products such as dairy products, cereals, fruits, or vegetables. The antifungal activity of *L. sakei* isolates from kimchi has been studied. A strain active against *Penicillium brevicompactum* through the production of organic acids has been reported [81]. *L. sakei* was shown to inhibit the growth of *Colletotrichum gloeosporioides*, *Botrytis cinerea*, *Penicillium expansum*, and *Aspergillus flavus*, but the inhibiting molecule(s) were not clearly identified [82].

## 6. *L. sakei*: The Bad Boy

As *L. sakei* is a psychrotrophic and facultative anaerobic species, it is favored by oxygen-depleted environments or vacuum packaging and cold temperature, conditions prevalent in meat product storage. Consequently it has also often been identified in large amounts in spoiled products [83], even if its role as a spoilage organism has not systematically been evidenced [84]. It should be also noticed that besides the *arc* operon responsible for arginine utilization through the arginine deiminase pathway, some strains harbor a second operon encoding the enzymes of an agmatine deiminase pathway [28]. The production of putrescine from agmatine has been demonstrated in these strains [28], and this may contribute to a spoilage potential of such *L. sakei* strains. Another concern is the carriage of antibiotic resistance genes, in particular when present in transmissible genetic elements, a property that does not fit with the necessity of starter or protective strains to be added in human food. Nevertheless the presence of acquired genes for resistance to clinically relevant antimicrobials as tetracycline and erythromycin resistant genes has been reported in *L. sakei* isolated from meat or pigs [85,86,87,88].

## 7. Conclusions

The species name given to *L. sakei* originates from its initial isolation and characterization from the fermented rice beverage sake. Despite this initial discovery from plant material, many subsequent studies reported its importance within the meat environment, even if it is nowadays isolated from other environments. The species undoubtedly possesses many fitness traits that ensure it an optimal growth and the capacity to survive under the conditions encountered during meat storage and fermentation. This probably explains its wide utilization as a meat starter. Thanks to many studies, the metabolic activities associated to its role as starter and its behavior during meat storage have been deeply investigated. One of the major traits of *L. sakei* is its high intra-species genetic diversity. Most of the knowledge that has been acquired results from experiments conducted with natural isolates selected by the scientific teams as “model” strains and then investigated mainly under laboratory conditions. It has nevertheless been reported that several strains coexist in meat matrixes [8], and that strain behavior may vary depending on the other strains present when co-cultured [41]. Furthermore, the use of strain combinations, in particular for biopreservation purpose, have been reported [64,65,66,67,68]. Taking into account the large intra-species diversity and the natural occurrence of several *L. sakei* strains sharing the same meat environment, one can propose that future trends should consider the species itself, with a core- and a pan-genome as well as a “core- and a pan-behavior”, rather than focusing only on individual strains. Indeed, the capacity of the species to overgrow other meat contaminants, either for fermentation or biopreservation use, certainly results from the sum of several selective advantages of several individuals behaving together as a cohort. The development of microbial ecology methods adapted to food environments should make it possible to investigate the roles of *L. sakei* as such, i.e., as a mixture of several strains acting together in a complex environment for the success of the species. This could also be extrapolated to understand the behavior of the species within environments hosting complex microbial communities, for improving its use as starter, developing biopreservation, and to understand why *L. sakei* may sometimes act as a spoilage bacterium.
